# Blood-Letting in Tarantula Bite

**Published:** 1889-09

**Authors:** T. J. Heard

**Affiliations:** Galveston


### BLOOD-LETTING FOR TARANTULA BITE.

GALVESTON, Sept. 5, 1889.

*Editor Daniel's Texas Medical Journal:*

In the August number of your JOURNAL, page 55, Dr. J. C. Baird reports a case of tarantula bite. This brings to my mind some of my own experience. In 1843, in the month of September, about 2 a. m., I was called to Dandridge Bibb in great haste.

He was abont twenty-five years of age, rather stout. I found him suffering the greatest torture and in a state of almost perfect collapse. About midnight he had been stung by a tarantula on his chest, and from thence the pain irradiated toward his spine, on reaching which point the pains became general and of the most agonizing character, He had scarcely any pulse at the wrist, and his breathing was very difficultr I plied him liberally with ammonia, camphor, morphine and sulphuric ether, without any effect, and I saw he was bound to die very soon, unless relieved. I reasoned thus: His blood is out of proportion to his powers of vital resistance, hence I will bleed him. With difficulty I raised a vein, made a good, free orifice, but the blood would not flow, only as I kneaded it away (that is for some time.) So soon as the blood flowed in a stream the poor fellow exclaimed: "Thank God! I feel it is going out with the blood." In twenty minutes he was entirely easy and went to sleep. The above, with Cook's pills given the next day, simply as a purgative, constitued all the treatment. He at once recovered.

T. J. HEARD, M. D.

